# Antiviral treatment for Bell’s palsy (idiopathic facial paralysis)

**DOI:** 10.1590/1516-3180.20151334T1

**Published:** 2015-08-03

**Authors:** Ildiko Gagyor, Vishnu B. Madhok, Fergus Daly, Dhruvashree Somasundara, Michael Sullivan, Fiona Gammie, Frank Sullivan

## Abstract

**BACKGROUND:**

Corticosteroids are widely used in the treatment of idiopathic facial paralysis (Bell’s palsy), but the effectiveness of additional treatment with an antiviral agent is uncertain. Significant morbidity can be associated with severe cases of Bell’s palsy.

**OBJECTIVES:**

To assess the effects of antiviral treatments alone or in combination with any other therapy for Bell’s palsy.

**METHODS:**

*Search methods:* On 7 October 2014 we searched the Cochrane Neuromuscular Disease Group Specialized Register, CENTRAL, MEDLINE, EMBASE, LILACS, DARE, NHS EED, and HTA. We also reviewed the bibliographies of the identified trials and contacted trial authors and known experts in the field and relevant drug companies to identify additional published or unpublished data. We searched clinical trials registries for ongoing studies.

*Selection criteria:* We considered randomised controlled trials or quasi-randomised controlled trials of antivirals with and without corticosteroids versus control therapies for the treatment of Bell’s palsy. We excluded trials that had a high risk of bias in several domains.

*Data collection and analysis:* Pairs of authors independently assessed trials for relevance, eligibility, and risk of bias, using standard Cochrane procedures.

**MAIN RESULTS:**

Eleven trials, including 2883 participants, met the inclusion criteria and are included in the final analysis. We added four studies to the previous review for this update. Some of the trials were small, and a number were at high or unclear risk of bias. Other trials did not meet current best standards in allocation concealment and blinding.

*Incomplete recovery:* We found no significant benefit from adding antivirals to corticosteroids in comparison with corticosteroids alone for people with Bell’s palsy (risk ratio (RR) 0.69, 95% confidence interval (CI) 0.47 to 1.02, n = 1715). For people with severe Bell’s palsy (House Brackmann scores of 5 and 6 or the equivalent in other scales), we found a reduction in the rate of incomplete recovery at month six when antivirals plus corticosteroids were used (RR 0.64, 95% CI 0.41 to 0.99, n = 478). The outcome for the participants receiving corticosteroids alone was significantly better than for those receiving antivirals alone (RR 2.09, 95% CI 1.36 to 3.20, n = 1169). The treatment effect of placebo was significantly lower than that of antivirals plus corticosteroids (RR 0.56, 95% CI 0.41 to 0.76, n = 658). Antivirals alone had a non-significant detrimental effect on the outcome compared with placebo (RR 1.10, 95% CI 0.87 to 1.40, n = 658).

*Motor synkinesis or crocodile tears:* In three trials comparing antivirals and corticosteroids with corticosteroids and placebo that assessed this outcome, we found a significant difference in long-term sequelae in favour or antivirals plus corticosteroids (RR 0.73, 95% CI 0.54 to 0.99, n = 869). Three trials comparing antivirals alone with corticosteroids alone investigating this outcome showed fewer sequelae with corticosteroids (RR 1.44, 95% CI 1.11 to 1.85, n = 873). We found no data on long-term sequelae for other comparisons.

*Adverse events:* Adverse event data were available in three studies giving comparison data on 1528 participants. None of the four comparisons (antivirals plus corticosteroids versus corticosteroids plus placebo or no treatment; antivirals versus corticosteroids; antivirals plus corticosteroids versus placebo; antivirals versus placebo) showed significant differences in adverse events between treatment and control arms. We could find no correlation with specific treatment within these results.

**AUTHORS’ CONCLUSIONS:**

Moderate-quality evidence from randomised controlled trials showed no additional benefit from the combination of antivirals with corticosteroids compared to corticosteroids alone or with placebo, and no benefit from antivirals alone compared to placebo, for the treatment of Bell’s palsy. Moderate-quality evidence showed a small but just significant benefit of combination therapy compared with corticosteroids alone in severe Bell’s palsy. We found no significant increase in adverse events from the use of antivirals compared with either placebo or corticosteroids.

**The full text of this review is available free of charge from:** http://onlinelibrary.wiley.com/doi/10.1002/14651858.CD001869.pub5/pdf/abstract.
